# Artificial Intelligence in Surgical Education: Considerations for Interdisciplinary Collaborations

**DOI:** 10.1177/15533506211059269

**Published:** 2021-12-10

**Authors:** Elif Bilgic, Andrew Gorgy, Meredith Young, Samira Abbasgholizadeh-Rahimi, Jason M. Harley

**Affiliations:** 1 Department of Surgery, 5620 McGill University, Montreal, QC, Canada; 2 Institute for Health Sciences Education, 12367 McGill University, Montreal, QC, Canada; 3 Department of Family Medicine, 12367 McGill University, Montreal, QC, Canada; 4 Department of Electrical and Computer Engineering, McGill University, Montreal, Canada; 5 Lady Davis Institute for Medical Research, Jewish General Hospital, Montreal, Canada; 6 Mila Quebec AI Institute, Montreal, Canada; 7 Steinberg Centre for Simulation and Interactive Learning, McGill University, Montreal, QC, Canada; 8 Research Institute of the McGill University Health Centre, Montreal, QC, Canada; 9 Department of Educational and Counselling Psychology, McGill University, Montreal QC, Canada

Artificial intelligence (AI) based devices are currently being used in the delivery of surgical care in a variety of settings.^1,2^ However, AI-enabled systems can trigger a variety of opinions and emotions, which reveals the different lenses that shape views on AI. Nonethless, work within surgical education may necessitate a more balanced view; with an acknowledgment of the participation of AI-enhanced devices in the delivery of surgical care and education, and consideration for how individuals can best utilize and complement these tools.^3^

In order to support a nuanced discussion of AI in surgical care, we can explore how AI has been reported and discussed in the literature. Unfortunately, the literature defining and describing AI is fragmented; likely contributing to the diverse views and reactions to AI-enabled technology. Hence, this editorial will synthesize a variety of definitions from various AI-adjacent fields in order to provide a brief and accessible definition of AI for surgical education, promoting and supporting dialogue amongst disciplines based on a shared understanding.

Based on examining definitions in *computer science/engineering*, AI is commonly defined as intelligent agents (ie system/program that can learn, adapt, change, and respond based on the inputs received from their environment) that act to achieve the best possible outcome, focusing on optimization of uncertain answers.^4^ As 1 example, AI-enabled alogrithm results in Google “predicting” what webpage would be most relevant to your search. In *Kindergarden-12 and general higher education*, AI is commonly defined as a computer-based learning system that performs tasks traditionally fulfilled by educators or tutors at an equivalent or superior level, adapting to the needs and goals of learners while providing personalized feedback.^5^ Examples include personal and collaborative tutoring systems that provide immediate, user-adaptive feedback to students. In *health care***,** AI is commonly defined as a branch of engineering and computer science which creates systems programmed to function and replicate intelligent human behavior, analyze complex medical data, assist physicians in diagnosing diseases, assist surgeons during a procedure, develop drugs along with treatment plans, predict patient outcomes, and determine optimal resource allocation.^6^

By drawing on the above definitions, we suggest the following definition for AI in surgical education: *An intelligent system/program that acts to fulfill or support the fulfillment of educational tasks traditionally performed exclusively by Surgical Educators, through making decisions in a manner similar to educators and providing customized adaptation, including performance assessment and feedback, to surgical trainees.*

With this definition in mind, we also suggest that AI systems used in surgical education can be classified in a variety of ways. The most straightforward means is to consider the core of how the AI system is programmed to work: with or without explicit rules. *Rule-based AI* generates pre-defined outputs based on certain rules programmed by humans, exhibiting ‘fixed intelligence’ by following a series of rules and instructions in a tree of steps (eg, decision tree). For example, an intelligent tutoring system developed to teach and assess cognitive and procedural skills required in basic robotic suturing, that uses interactive conversation trees and changes the content that students progress through (based on a baseline knowledge test and knowledge checks along the way) is an example of a rule-based AI system.^7^ On the other hand*, non-rule-based AI* simulates human intelligence to perform tasks without necessarily relying on pre-determined rules, building algorithms that automatically adapt to change, and this is the most popular application of AI in surgery and surgical education.^8,9^ For example, using supervised machine learning algorithms (artificial neural networks) to classify patterns of performance that distinguish between expertise levels in a virtual reality Sim-Ortho simulation task is an example of a non-rule-based AI.^10^

In conclusion, our definition contributes to building a shared understanding of AI in surgical education, which is an important step in building and strengthening bridges between and across disciplines, to enhance the development and application of AI into surgical training and associated research. Currently, progress is being made to develop AI-enhanced surgical education tools, such as virtual simulations that allow for personalized, flexible, and dynamic learning environments that would not be otherwise possible. As with any educational tool or technology, AI can be a powerful component of our eduational arsenal, when used purposefully and embedded thoughtfully within educational programs.
